# Region-specific protective effects of monomethyl fumarate in cerebellar and hippocampal organotypic slice cultures following oxygen-glucose deprivation

**DOI:** 10.1371/journal.pone.0308635

**Published:** 2024-08-07

**Authors:** Oliver Althaus, Nico ter Jung, Sarah Stahlke, Carsten Theiss, Jennifer Herzog-Niescery, Heike Vogelsang, Thomas Weber, Philipp Gude, Veronika Matschke

**Affiliations:** 1 Department of Cytology, Medical Faculty, Institute of Anatomy, Ruhr University Bochum, Bochum, Germany; 2 Department of Anesthesiology and Intensive Care Medicine, St. Josef Hospital, Ruhr University Bochum, Bochum, Germany; Drexel University, UNITED STATES OF AMERICA

## Abstract

To date, apart from moderate hypothermia, there are almost no adequate interventions available for neuroprotection in cases of brain damage due to cardiac arrest. Affected persons often have severe limitations in their quality of life. The aim of this study was to investigate protective properties of the active compound of dimethyl fumarate, monomethyl fumarate (MMF), on distinct regions of the central nervous system after ischemic events. Dimethyl fumarate is an already established drug in neurology with known anti-inflammatory and antioxidant properties. In this study, we chose organotypic slice cultures of rat cerebellum and hippocampus as an *ex vivo* model. To simulate cardiac arrest and return of spontaneous circulation we performed oxygen-glucose-deprivation (OGD) followed by treatments with different concentrations of MMF (1–30 μM in cerebellum and 5–30 μM in hippocampus). Immunofluorescence staining with propidium iodide (PI) and 4′,6-diamidine-2-phenylindole (DAPI) was performed to analyze PI/DAPI ratio after imaging with a spinning disc confocal microscope. In the statistical analysis, the relative cell death of the different groups was compared. In both, the cerebellum and hippocampus, the MMF-treated group showed a significantly lower PI/DAPI ratio compared to the non-treated group after OGD. Thus, we showed for the first time that both cerebellar and hippocampal slice cultures treated with MMF after OGD are significantly less affected by cell death.

## Introduction

Even in the present era, cerebral damage after cardiac arrest remains a significant challenge for which effective pharmacological interventions for neuroprotection are not yet available. In the United States alone, more than 350,000 individuals are affected by out-of-hospital cardiac arrest each year, with a survival rate of approximately 10.5% and as low as 8.5% with good functional status [1]. Moreover, a significant proportion of patients suffer from long-term neurological deficits, which in turn reduces their quality of life. A longitudinal study showed that 57% of patients who experienced out-of-hospital cardiac arrest exhibited a poor neurologic outcome one month after the event [2]. In addition to the loss of quality of life, patients with brain damage also incur substantial economic costs [3].

In a mere one minute, patients who suffer an acute ischemic stroke of the large vessels lose 1.9 million neurons and 14 million synapses [4]. This loss occurs minute by minute until the start of a treatment. When one considers the oxygen consumption of a resting body, the human brain alone consumes approximately 20% of the oxygen consumed [5]. It has been shown that neurons consume approximately 75–80% of the energy, while the rest is consumed by astrocytes and other cells [6, 7]. Consequently, a continuous blood supply is essential for the brain to ensure the delivery of sufficient oxygen and glucose. In general, after approximately four min without perfusion, as in the case of cardiac arrest, the ATP reserves in the brain are exhausted [8]. The cell membrane will depolarize and molecular mechanics, like the release of excitatory amino acids and Ca^2+^ overload, will commence [9]. The loss of function of the calcium pump due to ATP depletion, on the one hand, and the increased permeability of the membrane for calcium ions on the other hand, will result in an increase of the intracellular calcium concentration [10]. The influx of calcium ions results in glutamate excitotoxicity, and an influx of sodium ions also causes water to flow into the neurons, resulting in swelling [11, 12]. This calcium overload triggers several mechanisms leading to cell death [12–14]. Calcium ions also activate phospholipase A_2_ and cyclooxygenase, resulting in the generation of free radicals and other reactive oxygen species (ROS). These ROS lead to cell membrane damage and lipid peroxidation [9, 15]. Unfortunately, further reperfusion injury occurs after return of perfusion, as in a return of spontaneous circulation (ROSC). This appears to be caused by reperfusion-induced metabolism of free arachidonic acid, which is a source of ROS [16]. Mitochondrial dysfunction also appears to play an important role in ROS production [17–19]. ROS play a significant role in brain injury by damaging proteins, DNA, and lipids, causing necrosis or inducing cell death pathways [20–22]. This, in turn, leads to the expression of proinflammatory genes, which in turn amplifies the extent of brain damage [9, 23].

Due to the extremely limited neurogenesis in the adult human brain, which has only been described in the striatum [24] and the hippocampus [25], neuroprotection strategies are highly relevant. Apart from general intensive care measures, no specific neuroprotective strategies are currently known, apart from moderate hypothermia [26–29].

As previously stated, in addition to inflammatory processes, oxidative stress is also involved in ischemia-induced brain damage. Therefore, drugs with known antioxidant effects that have already been established in neurology will be investigated for their neuroprotective properties after circulatory arrest. In this study, the drug dimethyl fumarate (DMF), which has already been established in the treatment of multiple sclerosis [30, 31], is being examined. Within the gastrointestinal tract, DMF is metabolized to monomethyl fumarate (MMF), which is the active metabolite of DMF [32]. In cells, MMF binds in the nucleus to transcription factors such as nuclear factor erythroid 2-related factor 2 (NRF2), promoting the transcription of antioxidant genes which will reduce oxidative stress and cell death [33]. These genes include glutamate-cysteine ligase catalytic subunit (*GCLC*) and glutathione peroxidase 2 (*GPX2*) [34], heme oxygenase 1 (*HMOX1*) [35, 36], and glucose-6-phosphate dehydrogenase (*G6PD*) [37]. However, NRF2 independent mechanisms have also been described previously [38, 39].

The purpose of this study was to examine the protective effects of the active ingredient in dimethyl fumarate, MMF, on various areas of the central nervous system following ischemic incidents. To simulate the typical brain damage of cardiovascular arrest, the established model of oxygen-glucose-deprivation (OGD) in organotypic slice culture of cerebellum and hippocampus as an *ex vivo* model was used. This is followed by treatment of the slice cultures with MMF for 24 h.

## Methods

### Organotypic slice cultures

All procedures were conducted in accordance with the established standards of the German federal state of North Rhine Westphalia, in compliance with the European Communities Council Directive 2010/63/EU on the protection of animals used for scientific purposes. According to current German and European legislation, the removal of organs or cells from vertebrates for scientific purposes is not considered an animal experiment if the animals have not been subject to surgical interventions or invasive treatments prior to sacrifice. Consequently, the euthanasia of rat intended for the removal of brain tissue does not necessitate the approval or permission of local or governmental authorities.

For this study, organotypic slice cultures from rat cerebellum and hippocampus were used as previously described by Wolters and Reuther et al. [40]. In brief, at least six neonatal Wistar rats were decapitated by guillotine on postnatal day 9 (p9) without prior anesthesia. The Cerebellum and hippocampus were carefully dissected under strictly sterile conditions and stored in sterile ice-cooled Hank’s solution (#H8264-500ML; Sigma-Aldrich, Darmstadt, Germany). Using a McIlwain tissue chopper, the cerebellum was sliced along its sagittal axis into 275 μm slices, and the hippocampus was sliced along its long axis into 350 μm slices. The slices were subsequently transferred to cell culture inserts with semi-permeable membranes made of PTFE film (Millicell Cell Culture Inserts, 0.4 μm pore-size, #PICM0RG50, Merck, Darmstadt, Germany). The inserts are placed in six-well plates containing one ml of culture medium and are pre-incubated at 37°C and 5% CO_2_ for at least two h. The culture medium consists of Dulbeccos Modified Eagle Medium (DMEM; #A14430-01, Thermo Fisher Scientific, Waltham, MA, USA) containing 25% heat-inactivated horse serum (#16050–122; Thermo Fisher Scientific), 25% Hank’s balanced salts solution (NaCl 8 g/L, KCl 0.4 g/L, Na_2_HPO_4_ 0.048 g/L, KH_2_PO_4_ 0.06 g/L, CaCl_2_ 0.185 g/L, MgSO_4_ 0.098 g/L, NaHCO_3_ 0.35 g/L, glucose 1 g/L), 6.5% glucose, 2.5 mg/l NGF (N-0513; Sigma-Aldrich), 1% GlutaMax (#35050–061; Thermo Fisher Scientific), 1% PenStrep (10,000 U penicillin, 10 mg streptomycin, #P4333, Sigma-Aldrich), and 10 mg/l phenol red (#P0290; Sigma-Aldrich). The organotypic slice cultures were maintained at 37°C and 5% CO_2_ for seven days. Every other day, half of the cell culture medium was replaced with fresh cell culture medium [40].

### Oxygen-glucose-deprivation

After seven days of cultivation of the slice cultures, an OGD was conducted. The seven-day waiting period was chosen to allow the tissue slices to stabilize, which typically occurs between 5- and 20-days *ex vivo*. This period ensures recovery from initial cellular injury caused by the preparation process, resulting in more consistent and reliable experimental conditions. Additionally, the stabilization period enhances drug penetration, crucial for accurate MMF exposure. The OGD was performed in a humidified hypoxic chamber (O2 Control InVitro Glove Box; Coy Lab, Grass Lake, MI, USA) with an atmosphere of 0.4% O_2_, 5% CO_2_ and 37°C was used. The slice cultures were transferred to the hypoxic chamber and the glucose-containing medium was replaced with a glucose-free OGD medium prepared like the cell culture medium described above but without glucose, which had already been gassed for two h in the hypoxic atmosphere to assume oxygen-free conditions. The samples remained in the hypoxic chamber for 30 min for OGD. This shorter OGD duration was chosen to closely mimic transient ischemic events often encountered in clinical settings and to evaluate the potential of MMF under these conditions. OGD was terminated by removing the slice cultures from the hypoxic chamber thereby returning them to a normoxic atmosphere and transferring them back to fresh glucose-containing medium. Additionally, propidium iodide (PI, #P4170; Sigma-Aldrich) was added to the culture medium as an indicator of total cell death at a concentration of 5 μl/ml. At the same time, a control group was tested under normoxic conditions in the same OGD medium but with 6.5% glucose, stored for 30 min in an incubator at 5% CO_2_ and 37°C.

### Treatment with monomethyl fumarate

First, monomethyl fumarate (#651419, Sigma-Aldrich) was dissolved in dimethyl sulfoxide (DMSO, #7033; J.T.Baker, Fisher Scientific, Schwerte, Germany) to prepare a 50 mM stock solution and stored at -20°C. Immediately after transfer of the slice cultures into the normoxic and normoglycemic culture medium, treatment with MMF was performed. For this purpose, different concentrations of the compound (1.0, 3.0, 4.0, 5.0, 10.0, 12.5, 15.0, 17.5, 25.0 and 30.0 μM in cerebellum and 5.0, 6.0, 8.0, 9.0, 10.0, 12.5, 15.0, 17.5, 25.0 and 30.0 μM in hippocampus) were added to the culture medium. Our choice of concentrations has been guided by the results of previous studies of Parodi et al. [39] and Scannevin et al. [41]. Furthermore, PI was added to the medium at 1.0 μM to detect cell death. This was followed by 24 h of incubation. The slice cultures have been briefly washed with cooled phosphate buffer (PBS), before fixation with 4% paraformaldehyde (PFA) in PBS for 20 min at room temperature.

The following conditions were performed in one experiment: 1) control without OGD, without treatment; 2) control without OGD, with treatment (30 μM MMF); 3) OGD without treatment; 4) OGD with treatment (1.0, 3.0, 4.0, 5.0, 10.0, 12.5, 15.0, 17.5, 25.0 and 30.0 μM MMF in cerebellar slice cultures, respectively 5.0, 6.0, 8.0, 9.0, 10.0, 12.5, 15.0, 17.5, 25.0 and 30.0 μM MMF in hippocampal slice cultures). Given that the drug was dissolved in dimethyl sulfoxide (DMSO), it is necessary to ascertain whether DMSO has any effect on cells of the organotypic slice cultures under OGD and control conditions. For this purpose, the slice cultures were incubated with the maximum dose of DMSO (0.2%). Additionally, to ensure that MMF itself did not have a toxic effect on the cultures, we tested MMF at the highest concentration (30 μM). If no adverse effects are observed at this maximum concentration, it is reasonable to assume that lower concentrations would also be non-toxic.

For each concentration and condition, the experiment was performed a minimum of three times.

### Immunofluorescent staining and imaging

The immunostaining, imaging, and analysis were performed in accordance with the previously described methodology by Wolters and Reuther et al. [40]. The nuclei of the slice cultures were counterstained with 4′,6-diamidin-2-phenylindol (DAPI, #9542, Sigma-Aldrich). The slices were embedded on slides with Flouroshield (#F6937, Sigma-Aldrich) as mounting medium under coverslips and were stored in the dark. The slices were analyzed using a spinning disc confocal microscope (VisiScope Confocal-Cell Explorer, Visitron Systems GmbH, Puchheim, Germany). For this purpose, the full-size sections were acquired entirely via raster scan and z-stacking to include all cell layers during analysis. The images were acquired with a 20x magnification objective (Nikon PlanFluor 20×, NA 0.5; Nikon Instruments Europe BV, Amsterdam, Netherlands). The individual images were stacked and stitched to represent the entire specimen. Each image was captured with the same settings for exposure time and laser intensity to ensure reproducibility. To determine the total fluorescence signal, DAPI and PI images were processed using ImageJ 1.52q (National Institutes of Health, Bethesda, MD, USA) software. The DAPI images were first converted to 16-bit grayscale. Background noise was measured at multiple points and subsequently removed using the threshold command, ensuring that only DAPI-positive signals were preserved. The same procedure was applied to the PI images to isolate PI-positive signals. Both sets of images were then analyzed using the measure command in ImageJ. The total fluorescence intensity of PI (RawIntDent PI) was normalized to the total fluorescence intensity of DAPI (RawIntDent DAPI) to calculate the relative extent of cell death. This method allowed us to quantify the fluorescence signals accurately, considering the background noise and ensuring that only specific signals were measured. The analysis was performed on the entire section of each image, providing a comprehensive assessment of the fluorescence signals. Mean values of the OGD group without treatment were used for normalization and displayed as percentage. The values were plotted in a non-linear regression in order to determine the half maximal effective concentration (EC_50_) of MMF could be determined [40].

#### RNA-isolation, reverse transcription, and quantitative PCR

After the organotypic slice cultures were subjected to OGD and incubated with or without MMF (region specific EC_50_ concentration; cerebellum: 4.74 μM, hippocampus: 7.92 μM) for further 24 h, total RNA was isolated from four slices of cerebellum and six slices of hippocampus using the NucleoSpin miRNA Kit (#740971, Macherey-Nagel, Germany) according to manufacturer’s protocol. cDNA synthesis was performed with a reverse transcription system (#A3500, Promega, USA). Following the manufacturer’s protocol, 1 μg total RNA and oligo(dT) primer were used. The cDNA was stored at -20°C until use. Standardized quantitative real-time PCR was performed on a CFX96 Real-Time PCR Detection System (Bio-Rad, USA). GoTag qPCR Master Mix (#A6001, Promega, USA) was used with 50 ng cDNA and the corresponding primer sets (0.7 μM each). The following primer sequences were used: *Hmox1* forward 5’-GCC GAG AAT GCT GAG TTC AT-3’, reverse 5’-CTG CTT GTT GCG CTC TAT CT-3’; *Gpx2* forward 5’-AAT GTG GCG TCA CTC TGA GG-3’, reverse 5’-GGG AAG CCG AGA ACT ACC AG-3’; *G6pd* forward 5’-GGC AGC GGC AAC TAA ACT CA-3’, reverse 5’-CCT GGT ACA ACT CTT CCC TCA G-3’; *Gclc* forward 5’-ACA AGG ACG TGC TCA AGT GG-3’, reverse 5’-GTC TCA AGA ACA TCG CCT CCA-3’, *Actb* forward 5’-CTA AGG CCA ACC GTG AAA AG-3’, reverse 5’-AAC ACA GCC TGG ATG GCT AC-3’. Melting curves were recorded after each cycle and showed individual PCR products. Expression levels of the genes of interest and the housekeeping genes were measured in triplicate in three independent PCR runs.

### Statistical analysis

The data were analyzed statistically in accordance with the methodology previously described by Röderer et al. [42] and Wolters et al. [40]. GraphPad Prism 9 software (GraphPad Software, USA) was used for data evaluation. In brief, the information presented consists of the mean values obtained from a minimum of three independent experiments ± the standard error of the mean (SEM). A two-tailed Student’s t-test was used to test the significance of the data between two groups, while a one-way analysis of variance was used to compare data across multiple groups. Tukey’s multiple comparisons *post-hoc-test* was employed for making pairwise comparisons between groups. Results with a p-value less than 0.05 were deemed statistically significant [40, 42]. Given the high susceptibility of slices to damage from handling, the extent of which became apparent only during microscopy, the number of slices per concentration varied. In order to minimize the impact of scattering, the groups were pooled, and the mean value of each group was determined. For analyzing qPCR data, the collected data were analyzed using the 2^−ΔΔCT^ method and also the Kolmogorov-Smirnov normality test was used to confirm normal distribution.

## Results

### OGD induces cell death in organotypic slice cultures

**Fig 1:**
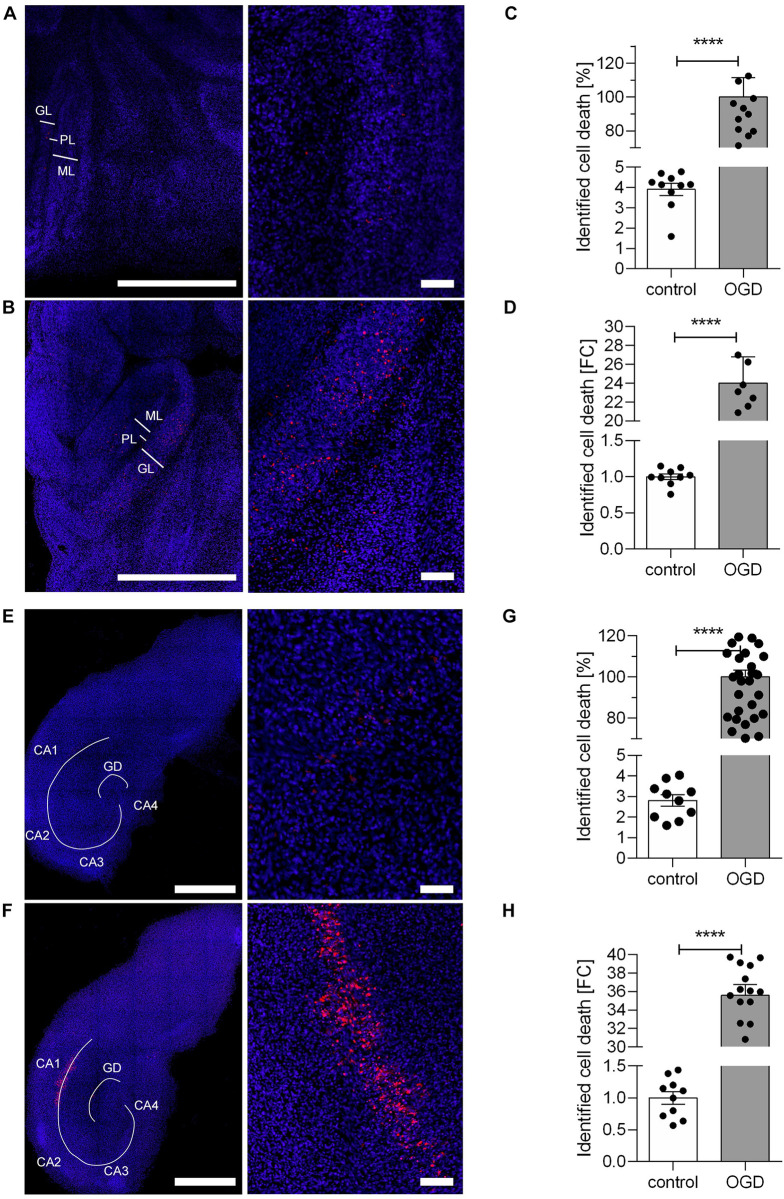
Cell death in cerebellar and hippocampal organotypic slice cultures after 30 min OGD. **A** The images illustrate the representative characteristics of cerebellar organotypic slice cultures following the control treatment. For nuclear staining DAPI (blue) was used. The presence of dead cells was identified by staining with PI (red). Left image: Scale bar: 1000 μm. Right image: Scale bar: 100 μm. **B** The images illustrate the representative characteristics of cerebellar organotypic slice cultures after following 30 min OGD. The staining procedure revealed an elevated PI signal in the rat cerebellum following 30 min of OGD. In the cerebellum, the PI signal was observed predominantly in the granular layer (GL) and to a less extent in the molecular layer (ML). Only a few positive signals for PI were observed in the Purkinje cell layer (PL) following OGD. For nuclear staining DAPI (blue) was used. The presence of dead cells was identified by staining with PI (red). Left image: Scale bar: 1000 μm. Right image: Scale bar: 100 μm. **C** The identification of cell death was conducted in organotypic cerebellar slice cultures in both the control and OGD groups. The PI signal was normalized to the DAPI signal. The slice culture group that followed OGD with no drug treatment was set at 100% for the proportion of identified cell death. **D** The calculation of identified cell death was conducted as a fold change [FC] to control conditions in the cerebellum. The PI signal was normalized to the DAPI signal. **E** The images illustrate the representative characteristics of hippocampal organotypic slice cultures following the control treatment. For nuclear staining DAPI (blue) was used. The presence of dead cells was identified by staining with PI (red). Left image: Scale bar: 1000 μm. Right image: Scale bar: 100 μm. A total of 9 to 26 slice cultures were analyzed per group, with a minimum of three independent preparations. **F** The images illustrate the representative characteristics of hippocampal organotypic slice cultures after following 30 min OGD. The staining procedure revealed an elevated PI signal in the rat hippocampus following 30 min of OGD. In the hippocampus, the PI signal exhibited a notable increase in the CA1 region and the dentate gyrus (DG). For nuclear staining DAPI (blue) was used. The presence of dead cells was identified by staining with PI (red). Left image: Scale bar: 1000 μm. Right image: Scale bar: 100 μm. A total of 9 to 26 slice cultures were analyzed per group, with a minimum of three independent preparations. **G** The identification of cell death was identified in the organotypic hippocampal slice cultures in both the control and OGD groups. The PI signal was normalized to the DAPI signal. The slice culture group that followed OGD with no drug treatment was set at 100% for the proportion of identified cell death. A total of 10 to 31 slice cultures were analyzed per group, with a minimum of three independent preparations. **H** The calculation of identified cell death was conducted as a fold change [FC] to control conditions in the hippocampus. The PI signal was normalized to the DAPI signal. A total of 10 to 31 slice cultures were analyzed per group, with a minimum of three independent preparations. All data are provided as mean ± SEM; ****p<0.0001 (two-tailed Student’s t-test).

The effect of hypoxia on organotypic slice cultures of the cerebellum and hippocampus was studied by evaluating and comparing the PI signal intensity as a marker for cell death of the different slice cultures. In the cerebellum, control slice cultures displayed diffuse and very low PI signals, which were sporadically distributed in a non-specific manner (**Fig 1A**). After 30 min of OGD followed by a 24 h reperfusion period, there was a marked increase in cell death. The PI signal was elevated, predominantly in the granular layer, with some isolated positive signals in the molecular layer (**Fig 1B**). In the cerebellar slice cultures, the percentage of identified cell death in the control group was approximately 4%, in comparison to the OGD group (**Fig 1C**), while the measured PI signal after OGD was 24 times higher compared to the control group (**Fig 1D**). These results, based on the PI/DAPI signal ratio, are highly statistically significant.

Hippocampal control slices showed a similarly low and diffuse PI signal (**Fig 1E**). However, following the OGD protocol, cell death was particularly pronounced in the hippocampus (**Fig 1F**). The PI signal was significantly increased in the CA1 region, while a less intense signal was noted in the CA3 region (**Fig 1F**). Quantitative analysis revealed that the control group showed around 3% cell death, compared to the OGD group (**Fig 1G**). The PI signal and inferred cell death in hippocampal slices were approximately 35 times higher after OGD compared to control conditions (**Fig 1H**).

### Monomethyl fumarate reduces cell death after OGD in cerebellar and hippocampal organotypic slice cultures

To determine whether MMF exhibits neuroprotective properties in organotypic slice cultures of the cerebellum and the hippocampus after induced ischemia, leading to a reduction in cell death, the drug was applied to the culture medium immediately after OGD and left for 24 h. To assess the EC_50_, different concentrations [μM] of the drug were tested: 1.0, 3.0, 4.0, 5.0, 10.0, 12.5, 15.0, 17.5, 25.0 and 30.0 in cerebellar slice cultures and 5.0, 6.0, 8.0, 9.0, 10.0, 12.5, 15.0, 17.5, 25.0 and 30.0 in hippocampal slice cultures.

**Fig 2:**
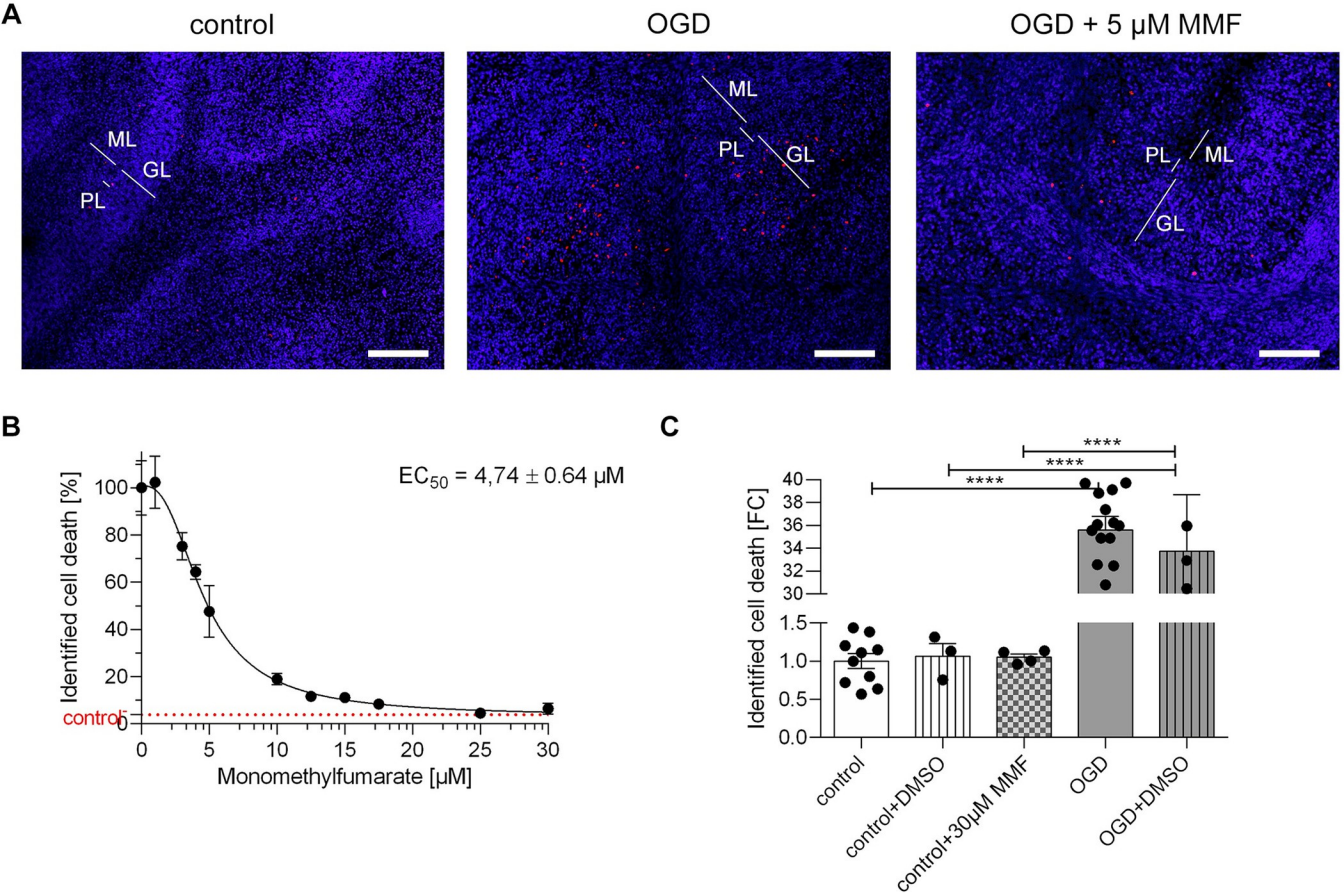
Effect of application of monomethyl fumarate in organotypic slice cultures of cerebellum after 30 min of OGD. **A** The images show an organotypic slice culture from the cerebellum, with an enlargement of the granular layer region. Illustrated from left to right are: The control group, the OGD group without treatment and the OGD group treated with MMF with the calculated EC_50_ of 4.74 ± 0.64 μM. An image of the test series with the corresponding concentration closest to the calculated EC_50_ has been selected as an example. Staining with DAPI marks all cell nuclei blue. Counterstaining with PI marks dead cells red. Treatment with MMF after 30 min of OGD results in significantly lower PI signal and thus significantly reduced cell death in the GL. Scale bars: 100 μm. **B** The graph shows the identified cell death after 30 min of OGD in percent plotted against the concentrations of MMF. In order to ascertain the percentage of cell death, the PI signal was normalized to the DAPI signal and expressed as a percentage. The slice culture group that followed OGD with no drug treatment was set at 100% for the proportion of identified cell death. The cell viability increased and equally the rate of identified cell deaths decreased with increasing concentration of MMF. All Data are provided as mean ± SEM; ****p<0.0001 (one-way analysis of variance with Tukey’s multiple comparisons *post-hoc-test*); the number of samples per group is n = 3–4, with at least three independent preparations. **C** The bars illustrate the identified cell death in organotypic cerebellar slice cultures under different control conditions. The PI signal was normalized to the DAPI signal. The identification of cell death was quantified as a fold change (FC) in comparison to the control conditions. The results demonstrated that the addition of 0.2% DMSO and the addition of 30 μM MMF did not lead to any significant difference regarding cell viability, when compared to the control group. Similarly, the addition of DMSO in the OGD group did not result in a significant difference in cell viability compared with the OGD group without DMSO. All Data are provided as mean ± SEM; ****p<0.0001 (one-way analysis of variance with Tukey’s multiple comparisons *post-hoc-test*); The number of samples per group is 10–26, with at least three independent preparations.

The effect of the drug on cell death in organotypic cerebellar slice cultures was analyzed by first determining the average intensity of the PI signal in the OGD group of cerebellar slice cultures that were not treated with the drug (**Fig 2A** middle image), which was defined as representing 100% cell death. Predominantly PI-positive signals were visible in the granular layer after a 30 min OGD. The average intensity of the PI signal for each cerebellar OGD group treated with the drug at different concentrations was then analyzed. These values were compared to the average PI signal intensity of the untreated OGD group to calculate the percentage of relative cell death.

The results showed a decrease in the PI signal, which previously appeared as diffuse conglomerates in the granular layer (**Fig 2A**, middle and right image). Notably, even at a concentration of 3 μM MMF, cell death is reduced by 24.8% (**Fig 2B**). As the concentration of MMF increased, the percentage of cell death decreased further. At a concentration of 25 μM, the effect reached the value of the control group. A further increase in concentration to 30 μM did not lead to an additional improvement in cell survival. The EC_50_ was subsequently calculated to be 4.74 ± 0.64 μM (**Fig 2B**).

Since MMF was dissolved in DMSO, and to exclude any influence of the solvent, DMSO at the highest concentration (0.2%) was used as a control. The application of DMSO did not result in a significant alteration in the ratio of PI/DAPI signal in cerebellar slice cultures in either the control group or the OGD group. To further ensure that MMF did not have a toxic effect in cerebellar slice cultures, MMF at the highest concentration used (30 μM) was added to the control group. The application of MMF at the highest concentration in the control group did not induce cell death or cell protection in slice cultures of the cerebellum (**Fig 2C**).

**Fig 3:**
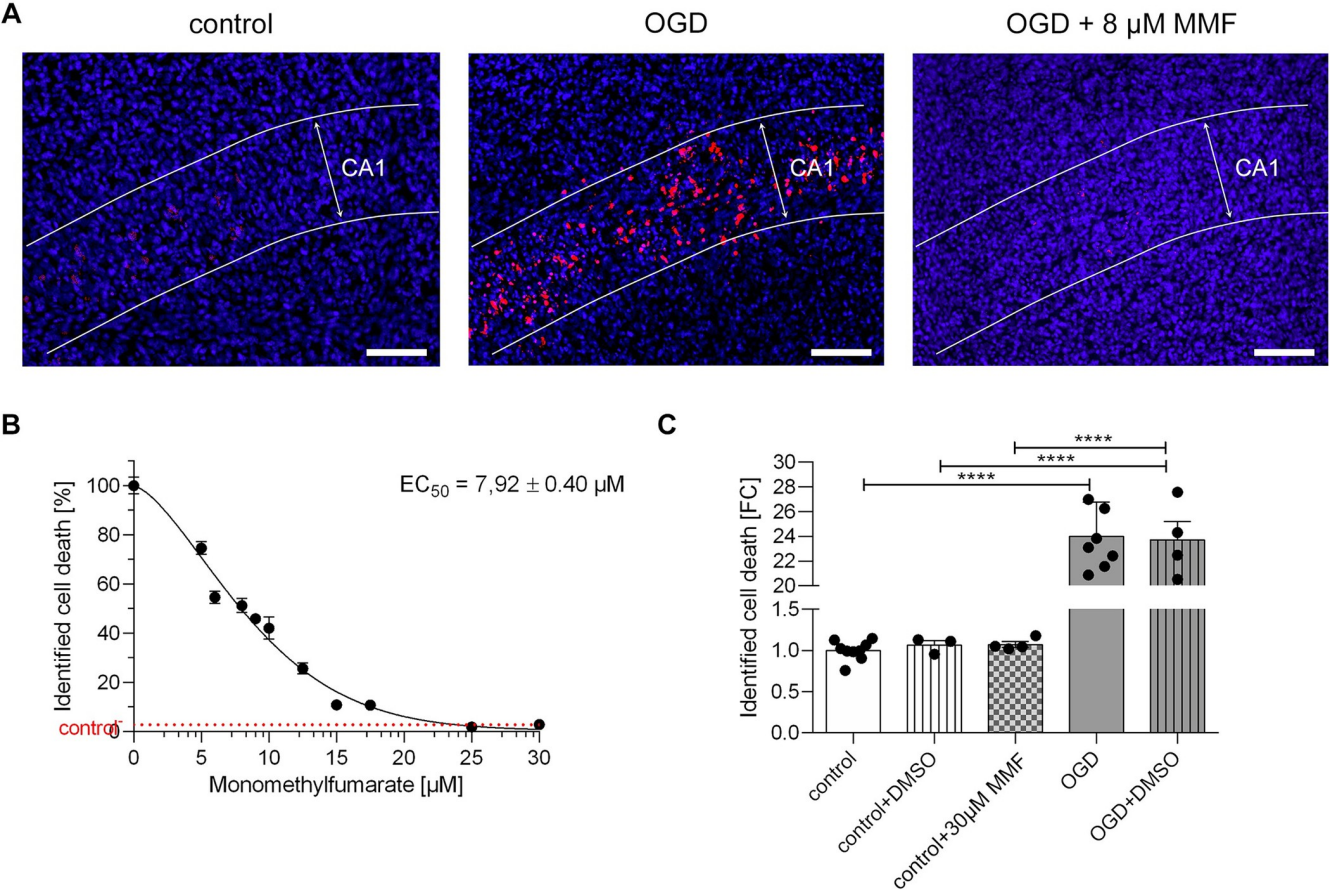
Effect of application of monomethyl fumarate in organotypic slice cultures of hippocampus after 30 min of OGD. **A** The images show an organotypic slice culture from the hippocampus, with an enlargement of the CA1 region. Illustrated from left to right are: The control group, the OGD group without treatment and the OGD group treated with MMF with the calculated EC_50_ of 7.92 ± 0.40 μM. An image of the test series with the corresponding concentration closest to the calculated EC_50_ has been selected as an example. Staining with DAPI marks all cell nuclei blue. Counterstaining with PI marks dead cells red. Treatment with MMF after 30 min of OGD results in a significantly lower PI signal and thus significantly reduced cell death in the CA1 region. Scale bars: 100 μm. **B** The graph shows the identified cell death after 30 min of OGD in percent plotted against the concentrations of MMF. In order to ascertain the percentage of cell death, the PI signal was normalized to the DAPI signal and expressed as a percentage. The slice culture group that followed OGD with no drug treatment was set at 100% for the proportion of identified cell death. The cell viability increased and equally the rate of identified cell deaths decreased with increasing concentration of MMF. All Data are provided as mean ± SEM; ****p<0.0001 (one-way analysis of variance with Tukey’s multiple comparisons *post-hoc-test*); the number of samples per group is n = 3–5, with at least three independent preparations. **C** The bars illustrate the identified cell death in organotypic hippocampal slice cultures under different control conditions. The PI signal was normalized to the DAPI signal. The identification of cell death was quantified as a fold change (FC) in comparison to the control conditions. The results demonstrated that the addition of 0.2% DMSO and the addition of 30 μM MMF did not lead to any significant difference regarding cell viability, when compared to the control group. Similarly, the addition of DMSO in the OGD group did not result in a significant difference in cell viability compared with the OGD group without DMSO. All Data are provided as mean ± SEM; ****p<0.0001 (one-way analysis of variance with Tukey’s multiple comparisons *post-hoc-test*); The number of samples per group is 10–31 with at least three independent preparations.

The effect of the drug on cell death in hippocampal organotypic slice cultures was analyzed in an in a manner analogous to the cerebellar slice cultures. This involved first determining the average intensity of the PI signal in the OGD group of hippocampal slice cultures that were not treated with the drug, which was defined as representing 100% cell death. These values were compared to the average PI signal intensity of the untreated OGD group to calculate the percentage of relative cell death. Predominantly PI-positive signals were visible in the CA1 region after a 30 min OGD (**Fig 3A**, middle image). The results showed a decrease in the PI signal, which was particularly evident in the CA1 region (**Fig 3A**, middle and right image). Notably, even at a concentration of 5 μM MMF, cell death was reduced by 25.4% (**Fig 3B**). In contrast to the cerebellum, a higher concentrations of the drug is required to elict an effect in the hippocampus. At a concentration of 25 μM, the effect reached the level of the control group. However, a further increase in concentration to 30 μM did not lead to an additional improvement in cell survival. The EC50 was subsequently calculated to be 7.92 ± 0.40 μM (**Fig 3B**). As observed in the cerebellar slice cultures, the addition of 0.2% DSMO did not result in a significant change in the ratio of PI/DAPI signal in hippocampal slice cultures, either in the control group or in the OGD group. Similarly, the addition of MMF at a concentration of 30 μM to the control group did not lead to any significant changes in cell death or cell protection (**Fig 3C**).

### Monomethyl fumarate region-specifically enhances gene expression after OGD treatment

**Fig 4:**
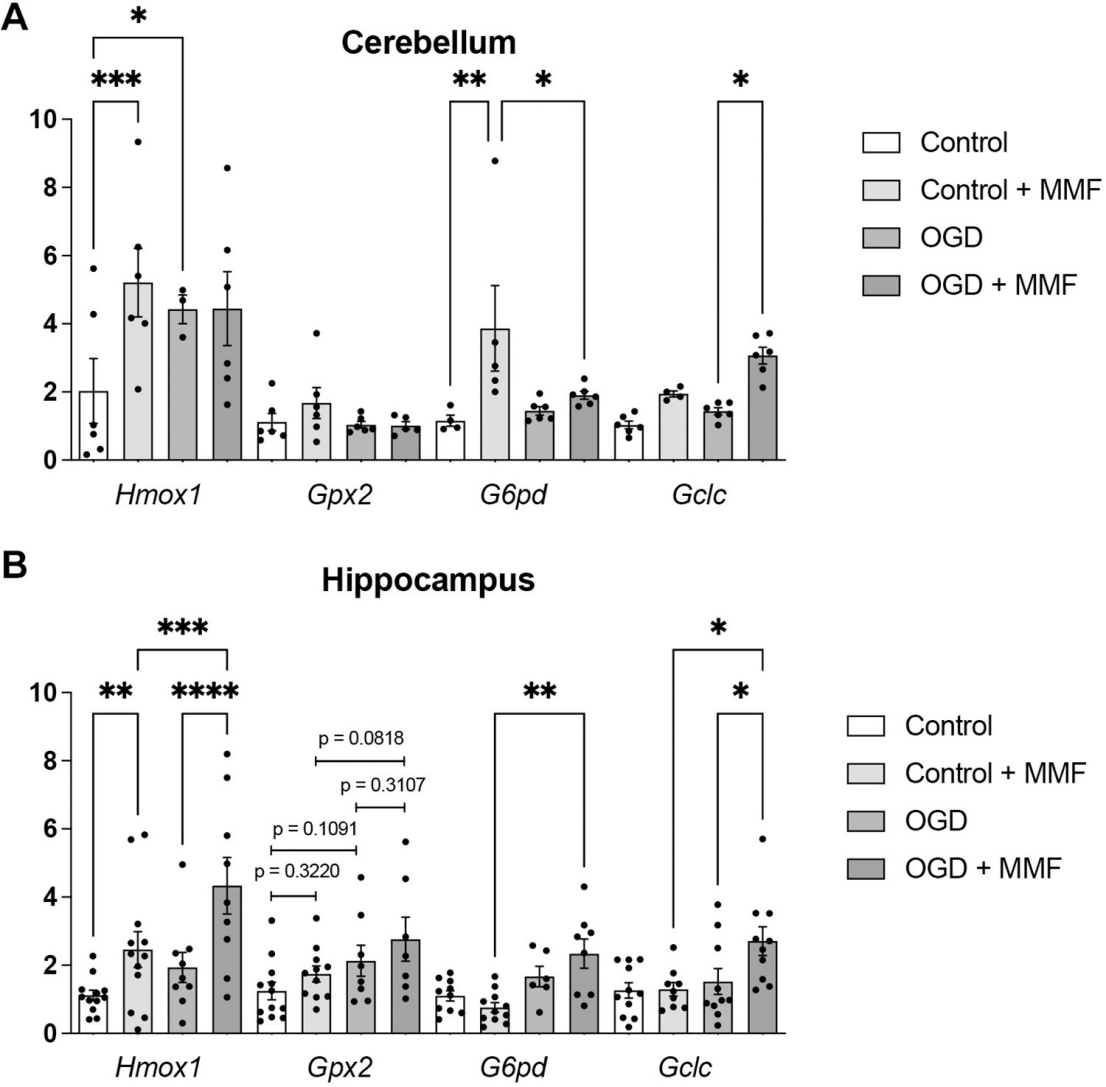
Effect on gene expression in organotypic slice cultures of the cerebellum and hippocampus after 30 min of OGD, using region-specific EC_50_ concentrations of MMF as determined for each brain region. **A** The mRNA expression levels in cerebellar slice cultures of heme oxygenase 1 (*Hmox1*), glutathione peroxidase 2 (*Gpx2*), Glucose-6-phosphate dehydrogenase (*G6pd*), and glutamate cysteine ligase catalytic subunit (*Gclc*) were investigated after OGD with and without treatment with MMF for 24 h by qPCR. **B** The mRNA expression levels of the appropriate genes in hippocampal slice cultures were investigated after OGD with and without treatment with MMF for 24 h by qPCR. For relative quantification, the 2^−ΔΔCt^ method was performed using actin *ACTB* for normalization. Gene expression was investigated in triplicates. FC = Fold Change; n = 4–6. N = 3. All data are provided as mean ± SEM. Data were tested for significance using Student‘s t-test. Significant differences are indicated by *p<0.05, **p<0.01, ***p<0.001, ****p<0.0001. For non-significant changes in *Gpx2* expression in hippocampal slice cultures, p-values have been included to illustrate the observed trend.

In order to gain insight into the mechanisms by which MMF treatment leads to a reduced proportion of dead cells following OGD, we conducted gene expression studies on specific genes known for their anti-oxidative properties using quantitative PCR. Our findings indicated that treating organotypic cerebellar slice cultures with MMF resulted in increased mRNA levels of *Hmox1* and *G6pd* (**Fig 4A**). In contrast, exposure to OGD for 30 min led to an increase in *Hmox1* 24 h post-OGD (**Fig 4A**). Upon examination of gene expression following OGD and 24 h incubation with MMF, it became evident that MMF application post-OGD did not result in a significant increased expression of the investigated genes (**Fig 4A**). In organotypic hippocampal slice cultures, the results were found to be differential (**Fig 4B**). Here, treating the cultures with MMF under normoxic conditions resulted in an increase of *Hmox1*. A 30 min OGD is insufficient to observe any change in gene expression 24 h post-OGD (**Fig 4B**). However, the use of MMF following a 30-minute OGD not only significantly increased the expression of *Hmox1* but also of *G6pd* and *Gclc*, along with a trend towards an increase, though not significant, in *Gpx2* (p = 0.0818; **Fig 4B**).

## Discussion

### Organotypic slice cultures and OGD as a model for simulating circulatory arrest in neuronal tissue

Endisch et al. demonstrated that the cerebellum and hippocampus are among the most vulnerable regions to severe brain damage following cardiac arrest. As a consequence, this study investigated these two regions [43]. In the present study, we selected organotypic slice cultures of cerebellum and hippocampus as model systems in this study because organotypic slice cultures from brain tissue are an established and suitable method to mimic the morphology and functionality, as well as the molecular processes, of brain tissue [44–47]. This is due to the preservation of the three-dimensional architecture of the tissue, which is a significant advantage over dissociated cell cultures [44, 46, 48]. Consequently, even vessels such as capillaries are preserved despite the absence of blood [49, 50]. Furthermore, this allows for interaction between all cell types that are physiologically present in the tissue [51]. However, despite these advantages, there are also some disadvantages. For instance, although the blood vessels are preserved, they no longer form a functional compartment [52]. This means that factors such as cerebrovascular autoregulation, intracranial pressure, or neurovascular coupling no longer have any influence [53]. Additionally, Morrison et al. have observed that mechanical stress on organotypic slice cultures can result in cell death. Therefore, it is crucial to exercise caution during the preparation and subsequent handling of these slice cultures [54]. However, since this phenomenon also occurs in our control group, these errors arising from mechanical stress during handling can be largely avoided. Another disadvantage is that organotypic slice cultures are only suitable for the observation of short-term effects of drugs since observation over a longer period is not possible. This is due to the fact that the slices cannot survive *ex vivo* for longer than three weeks and will perish within three days after severe injury [53]. Furthermore, OGD is a well-established and reliable model for simulating cerebral ischemia *ex vivo* [55–57], and our study again demonstrated that OGD reliably leads to cell death. The return of the slice cultures to normoxic and normoglycemic conditions also simulates reperfusion, as after a ROSC *in vivo*. This is a highly desirable outcome in our study, as it allows us to simulate the clinical setting of cardiac arrest.

### Inflammation and oxidative stress lead to cell death

Ischemic brain injury results from highly complex pathophysiological processes [58] and can be divided into early-onset primary injury, which occurs immediately after cardiac arrest and can be explained by ATP depletion and breakdown of ion channel function, and later-onset secondary injury, which includes reperfusion injury that occurs after ROSC [59]. It is now well known and described that both inflammation and oxidative stress play significant roles in ischemic brain damage caused by reperfusion injury [60–63]. For example, reperfusion injury leads to increased production and accumulation of ROS, which is one of the main reasons for cell damage after ischemia [64], whereupon damaged brain cells release inflammatory mediators such as tumor necrosis factor α (TNFα) or interleukin 1β (IL-1β) as well as adhesion molecules, triggering inflammation that is also part of the brain injury [9]. Adrie et al. even postulate that reperfusion after cardiac arrest leads to a sepsis-like syndrome with a systemic inflammatory response and high levels of circulating cytokines and adhesion molecules [65]. In their review, Jou et al. also highlighted the importance of cytokines in the post-cardiac arrest syndrome. In particular, IL-1β and TNFα increase substantially during reperfusion [66]. With this in mind, we decided to test a drug with known anti-inflammatory and antioxidant properties for neuroprotective effects after cardiac arrest. As slice cultures only represent an isolated organ without a fully functional systemic immune system, we initially considered the possibility that MMF might have an impact on oxidative stress via the NRF2 pathway in our ex vivo model, particularly due to hypoxia-reoxygenation. Since the migration of immune cells plays a significant role in cerebral damage [67], future research in animal models should include assessments of the anti-inflammatory effects of MMF, particularly in the context of hypoxia-reoxygenation-induced oxidative stress, to provide a more comprehensive understanding of MMF’s mechanisms of action and its therapeutic potential in clinical settings. In our experimental design, treatment was intentionally initiated during the reperfusion phase to simulate the treatment immediately after the occurrence of the event in humans, as it would occur in clinical practice. This allows the early-onset primary injury to occur unaffected by MMF, while the reperfusion injury occurs under the intended influence of MMF.

### Dimethyl fumarate shows neuroprotective effects

DMF is a drug approved in Europe by the EMA and in the U.S. by the FDA for the treatment of relapsing-remitting multiple sclerosis (MS). Its efficacy in humans with MS has been demonstrated in several clinical trials [30, 31]. In addition, potential beneficial effects have already been shown in other neurological disorders such as Huntington’s disease [68], stroke [69], and even traumatic brain injury [70]. DMF is metabolized to MMF in the gastrointestinal tract [32]. Therefore, we chose to use MMF. This is logical because MMF is the active metabolite in the brain and we are studying this direct effect in the brain.

It is hypothesized that both DMF and MMF exert their effects by activating the NRF2 pathway. This pathway plays a crucial role in cellular defense mechanisms against oxidative stress by inducing the production of antioxidant enzymes in neurons and astrocytes [33, 35, 38, 41, 71]. This induction is achieved by increasing the transcription of genes such as *GCLC*, *GPX2* [34], *HMOX1* [35, 36], and *G6PD* [37], all of which contribute to the antioxidant effects of NRF2. The results of our gene expression studies provide partial evidence for the activation of the NRF2 pathway under the experimental conditions we chose. Specifically, we observed a significant increase in *Hmox1* expression in the hippocampus following treatment with MMF at the EC_50_ concentration for 24 h under normoxic conditions. In the cerebellum, a similar treatment with MMF led to a significant increase in the expression not only of *Hmox1* but also of *G6pd*, highlighting the specific and differentiated response of different brain regions to the treatment.

In the context of ischemia reperfusion injury, a condition known to increase oxidative stress, elevated expression of NRF2 in neurons and astrocytes also leads to increased expression of these protective genes [72, 73]. However, in our experiments, we only observed a significant increase in *Hmox1* expression in the cerebellum, without further increases in the expression of the other genes investigated. This discrepancy may be partly due to the chosen OGD duration of 30 min, which may not be sufficient to induce a stronger activation of the NRF2 pathway. Notably, other studies that have observed NRF2 activation following OGD have employed a minimum OGD duration of 90 min [74–76]. This longer exposure likely provides a more pronounced stress signal necessary for robust NRF2 pathway activation and subsequent induction of its target genes. The specificity of NRF2 activation, as well as the temporal dynamics of gene expression in response to oxidative stress, suggests that the duration of OGD is a critical factor. Longer OGD exposure may be required to fully activate the NRF2 pathway and induce more pronounced expression of its target genes.

Our further gene expression analysis provides a more comprehensive overview of the neuroprotective effects of MMF under conditions of oxygen deprivation and reperfusion. Specifically, when analyzing the hippocampus, the comparison between the control + MMF and OGD + MMF groups reveals a remarkable divergence in gene expression patterns. Here, we observed a significant elevation in the levels of not only *Hmox1*, but also *G6pd* and *Gclc* enzymes. A minor, though not statistically significant, increase in *Gpx2* was also observed. This broader activation of NRF2 pathway genes in the hippocampus, despite the same OGD exposure duration, suggests a region-specific sensitivity to MMF treatment. This phenomenon may contribute to the enhanced cell viability in hippocampal slice cultures after MMF treatment after OGD.

However, when comparing control + MMF with OGD + MMF in cerebellar slice cultures, there was no significant elevation in these genes. This suggests that alternative mechanisms could be contributing to the enhanced cell viability observed in cerebellar slice cultures. Our experimental data revealed a differential sensitivity to MMF in cerebellar versus hippocampal tissues, as evidenced by a lower EC_50_ concentration for MMF in cerebellar tissues. This critical finding was incorporated into our methodology; we strategically utilized these EC_50_ values to determine the respective concentrations of MMF for treatment in our expression analysis. Consequently, cerebellar slices were exposed to comparatively lower concentrations of MMF. This differential dosing could potentially explain the varying effects observed between cerebellar and hippocampal slice cultures in gene expression studies. It is postulated that the reduced MMF concentration in the cerebellum may have resulted in lower alterations in gene expression, which may account for the divergent cellular responses observed in these cerebellar samples. These observations emphasize the possibility of alternative cellular mechanisms underlying the increased viability of cells in cerebellar slice cultures that differ from those in the hippocampus. While the role of MMF concentration in modulating gene expression is certainly significant, it is also crucial to consider the potential contribution of other NRF2 independent pathways to the observed effects.

However, other NRF2 independent mechanisms, such as a direct effect on inflammatory microglia, have also been previously discussed [38, 39]. Clausen et al. showed that six h after treatment, the concentration of IL-10 significantly increased, resulting in a downregulation of pro-inflammatory signaling cascades [38]. This can be further explained by the anti-inflammatory properties of IL-10, which suppress TNFα [77]. Lin et al. simulated a stroke caused by middle cerebral artery occlusion and demonstrated that treatment with DMF leads to a significant decrease in T-lymphocytes in the infarct area. This suggests that DMF acts as an immunomodulator, which cannot be explained by the NRF2 pathway alone [78]. Given these findings and the results from our gene expression analysis, further research is necessary on the mechanisms of neuroprotection of the cerebellum.

To our knowledge, this is the first demonstration that treatment with MMF during reperfusion leads to a reduction in cell death after OGD in both cerebellar and hippocampal slice cultures. This was evidenced by a significant signal reduction in the PI/DAPI ratio after cerebellar and hippocampal samples were treated with MMF after OGD compared to the untreated OGD group. However, we obtained different results between cerebellum and hippocampus. While the cerebellum can already benefit from very low concentrations of MMF, a half maximal effective concentration (EC_50_) is reached at 4.74 ± 0.64 μM, the hippocampus requires slightly higher concentrations so that the half maximal effective concentration (EC_50_) is only reached at 7.92 ± 0.40 μM. However, under the maximum concentration of MMF, both the cerebellum and the hippocampus exhibit values comparable to those of the untreated control group without OGD.

Thus, our study demonstrates differential sensitivity of cerebellar and hippocampal tissues to MMF, indicating the need for tissue-specific concentration adjustments to achieve optimal therapeutic outcomes. This differential sensitivity underscores the importance of tailoring therapeutic approaches based on the specific needs of each tissue type. Importantly, higher concentrations of MMF, which are beneficial for the hippocampus, were also found to be safe for the cerebellum. This suggests that therapeutic strategies could be developed to accommodate these differences, ensuring efficacy across different brain regions. However, it is crucial to consider the known lethal dose values of DMF or MMF for clinical use to ensure safety. DMF is currently approved as an oral formulation, which poses challenges for administration, especially in acute pathological conditions. In clinical practice, MMF/DMF is applied via a stomach tube. To address these challenges, future research should focus on conducting detailed pharmacokinetic and pharmacodynamic studies in animal models. These studies aim to refine the optimal concentrations of MMF/DMF required for different tissues and to determine the most effective administration routes. Assessing the systemic distribution and tissue-specific uptake of MMF will allow for the optimization of dosing regimens to enhance therapeutic benefits and minimize potential side effects. Future animal studies are expected to provide comprehensive data to guide the design of clinical trials and the development of protocols for the therapeutic application of MMF/DMF in various injury contexts.

## Conclusion

This study provides novel insights into the protective effects of MMF in organotypic cerebellar and hippocampal slice cultures following OGD, a model for the representation of ischemic brain damage like that which occurs during cardiac arrest. Our results demonstrate a significant reduction in cell death, as indicated by decreased PI/DAPI ratios, in both cerebellar and hippocampal cultures treated with MMF post-OGD. These findings suggest that MMF has potential as a therapeutic agent for reducing cellular damage following ischemic events.

Notably, our study revealed a differential response to MMF treatment between the cerebellar and hippocampal tissues, with cerebellar slices requiring lower concentrations of MMF to achieve protection. This difference could be attributed to distinct sensitivities and cellular mechanisms operating in these brain regions. The lower EC_50_ concentration in cerebellar tissues indicates a region-specific efficacy of MMF, emphasizing the importance of considering regional variability in brain responses to therapeutic interventions.

Furthermore, our analysis indicates that the protective effects of MMF may not be solely attributed to its action on the NRF2 pathway. The lack of significant elevation in NRF2 targeted genes in cerebellar slices treated with MMF suggests the involvement of alternative pathways, possibly including direct effects on inflammatory responses. This complexity highlights the need for further research to fully elucidate the multifaceted mechanisms of MMF’s protective effect.

In conclusion, our findings contribute valuable knowledge to the field of neuroprotection, particularly in the context of ischemic brain injury following cardiac arrest. The evident protective properties of MMF, along with its differential regional effects and potential involvement of NRF2-independent pathways, open new avenues for research and therapeutic strategies. Further studies, both *in vivo* and in clinical trials, are necessary to further explore the applicability of MMF in neuroprotective therapy and to optimize its therapeutic potential for diverse brain regions affected by ischemic injury.
